# Effect of pegylated interferon-α2b add-on therapy on renal function in chronic hepatitis B patients: A real-world experience

**DOI:** 10.3389/fmicb.2022.980250

**Published:** 2022-10-18

**Authors:** Mei-Juan Peng, Xiao-Qing Guo, Wei-Lu Zhang, Jing Chen, Wen Kang, Xiao-Fei Yang, Ying Guo, Ye Zhang

**Affiliations:** ^1^Department of Infectious Diseases, Tangdu Hospital, Fourth Military Medical University, Xi’an, China; ^2^Department of Hepatology, The Third People’s Hospital of Taiyuan, Taiyuan, China; ^3^Department of Epidemiology, School of Public Health, Fourth Military Medical University, Xi’an, China; ^4^Xiamen Amoytop Biotech Co., Ltd., Xiamen, China

**Keywords:** chronic hepatitis B, renal function, pegylated interferon-α2b, antiviral, mixed linear model

## Abstract

**Background and aim:**

Controversy remains as to pegylated interferon-α (PEG-IFNα) antiviral therapy to renal function in chronic hepatitis B (CHB) patients. The aim of this study was to evaluate the influence of PEG-IFNα2b (Y shape, 40 kD) add-on treatment for renal function in CHB patients who received entecavir therapy.

**Methods:**

This was a retrospective observational study to investigate factors related to renal function in 114 CHB patients who received PEG-IFNα2b add-on therapy to entecavir for 48 weeks. Changes of blood urea nitrogen (BUN), serum creatinine (sCr), and estimated glomerular filtration rate (eGFR), which was calculated with both Chronic Kidney Disease Epidemiology Collaboration and Modification of Diet in Renal Disease (MDRD) formulas, were analyzed by one-way analysis of variance. A linear mixed effects model for repeated measures was used to assess the correlation between baseline information and eGFR changes at 24 and 48 weeks of therapy. The model considered the baseline age, gender, body weight, viral load, hepatitis B surface antigen, BUN, sCr, and treatment strategy as fixed effects and incorporated random effects for individual subjects.

**Results:**

BUN and sCr was decreased, while eGFR was increased at 12 weeks of therapy. Only eGFR maintained at 24 and 48 weeks of therapy. Patients with female gender, age ≥ 40 years, and baseline HBsAg level < 250 IU/mL showed significant improvement of renal function with PEG-IFNα2b add-on therapy. The linear mixed effects model revealed that female gender, baseline sCr, and PEG-IFNα2b add-on were significant positive predictors for eGFR elevation at 24 and 48 weeks of therapy.

**Conclusion:**

In real-world experience, PEG-IFNα2b add-on therapy might be associated with increased eGFR in CHB patients.

## Introduction

Hepatitis B virus (HBV) infection is still a world-wide public health challenge. There is a close relationship between chronic hepatitis B (CHB) and chronic renal disease (Combes et al., 1971). HBV infection is associated with nephropathy, and renal dysfunction is frequently observed in patients with CHB and liver cirrhosis (Ren et al., 2019). HBV-induced impairment of renal function is mainly through deposition of immune complex in the kidney, leading to membranous nephropathy and mesangiocapillary glomerulonephritis (Gupta and Quigg, 2015; Shah and Amarapurkar, 2018). The potential risk factors contributing to renal dysfunction in CHB patients include elder age, co-infection with human immunodeficiency virus (HIV), pre-existing renal failure and comorbidities, hypertension, diabetes mellitus, end-stage liver diseases, and administration of nephrotoxic agents (Mallet et al., 2015; Rodriguez-Novoa et al., 2016; Yang and Choi, 2017; Zhang et al., 2017). Thus, it is pivotal to monitor renal function to avoid potential nephrotoxic effects before and during anti-HBV treatment by determination of blood urea nitrogen (BUN), serum creatinine (sCr), and estimated glomerular filtration rate (eGFR) (Ren et al., 2019).

HBV-associated renal diseases always improve through inhibition of viral replication mediated by antiviral treatments (Kupin, 2017). Therapeutic approaches for CHB include nucleos(t)ide analogs (NAs) and interferon-α. Six NAs are available for CHB treatment in China, including three nucleoside analogs [lamivudine (LAM), telbivudine (LdT), and entecavir (ETV)] and three nucleotide analogs [adefovir (ADV), tenofovir disoproxil fumarate (TDF), and tenofovir alafenamide (TAF)]. Treatment of CHB irrespective of medication, including LAM, ADV, ETV, and TDF, seems to result in a mild decrease of renal function (Mauss et al., 2011; Udompap et al., 2018), because the primary route of NAs elimination is renal excretion with unchanged drugs (Zhang et al., 2017). Patients receiving ADV and TDF therapy even experience more rapid loss in eGFR (Udompap et al., 2018). In contrast, several studies suggested that TDF is not associated with greater degree of kidney injury compared with other NAs (Trinh et al., 2019; Fischer et al., 2021) and untreated HBV-infected individuals (Wang et al., 2021). LdT has been demonstrated to improve renal function in CHB patients (Gane et al., 2014), in liver transplant recipients for HBV-related cirrhosis (Cholongitas et al., 2015), and in liver transplant recipients with long-term chronic kidney disease (Lee et al., 2017). However, the renal protective effect of LdT is still uncertain for hepatitis B surface antigen (HBsAg)-positive renal transplant recipients (Yang et al., 2020). Importantly, TAF reveals continuous improvement of kidney function in both treatment-naïve CHB (Agarwal et al., 2018) and TDF-experienced patients (Fong et al., 2019; Farag et al., 2021).

Pegylated interferon-α (PEG-IFNα) is also one of the first-line antiviral agents due to higher HBsAg loss, finite therapeutic duration, and absence of drug resistance (Charatcharoenwitthaya et al., 2021), leading to functional cure of CHB (Ning et al., 2019). Combination of PEG-IFNα and NAs therapy may improve the serological response, but remains controversial (Li et al., 2015; Bourliere et al., 2017). However, few studies focus on the safe renal profile of PEG-IFNα therapy in CHB patients. We previously showed that 48-week PEG-IFNα2a treatment revealed a renal protective effect for CHB patients (Zhang et al., 2017). In contrast, Su et al. (2018) showed that PEG-IFNα2b monotherapy or combined with ADV therapy did not cause further renal impairment. Herein, we aimed to assess the change of renal function under PEG-IFNα2b (Y shape, 40 kD) add-on therapy to ETV in CHB patients.

## Materials and methods

### Institutional review board

The study protocol was approved by Ethics Committee of Tangdu Hospital (Approval No. TDLL-201505-013) and Ethics Committee of the Third People’s Hospital of Taiyuan (Approval No. 2021-19). The study was conformed to the guidelines of the Declaration of Helsinki and the principles of Good Clinical Practice. Written consent was obtained from all patients, whose data were anonymized. The data were collected on April and May, 2022. We had access to information that could identify individual enrolled subjects during and after data collection.

### Study design

The retrospective observational cohort study was conducted. Inclusive criteria: (1) Diagnosis of CHB met the diagnostic standard of Chinese National Program for Prevention and Treatment of Viral Hepatitis; (2) Patients had received ETV therapy for more than 1 year; (3) HBV DNA level < 1,000 IU/mL. (4) HBsAg level < 1,500 IU/mL, because baseline HBsAg less than 1,500 IU/mL was associated with high rate of HBsAg loss (Hu et al., 2018; Ning et al., 2019; Wu et al., 2020). Exclusive criteria: (1) Co-infected with other hepatitis viruses or HIV; (2) Concurrently afflicted by end-stage liver diseases (including decompensated liver cirrhosis, severe hepatitis, liver failure, and hepatocellular carcinoma); (3) Afflicted by autoimmune disorders; (4) Afflicted by alcoholism or drug addiction; (5) Afflicted by solid cancers or leukemia. The enrolled patients received PEG-IFNα2b (Y shape, 40 kD; 180 μg, subcutaneous injection weekly; Xiamen Amoytop Biotech Co., Ltd., Xiamen, Fujian Province, China) add-on therapy to ETV (0.5 mg, orally once daily) for 24∼48 weeks between July 2018 and March 2022 in Tangdu Hospital or the Third People’s Hospital of Taiyuan. “Add-on” strategy was defined as addition of PEG-IFN to on-going NAs therapy in virally suppressed patients (Ning et al., 2019). The five observation time point was baseline, 12, 24, 36, and 48 weeks post add-on of PEG-IFNα2b. The blood samples were collected from each enrolled patients at least in three observation time points.

### Evaluation of virological index

Serum HBV DNA was quantified by real-time fluorescence quantitative polymerase chain reaction using a commercial HBV DNA detection (Xiamen Amplly, Xiamen, Fujian Province, China) with a detection limit of 50 IU/mL. HBsAg, anti-HBs, hepatitis B e antigen (HBeAg), anti-HBe was quantified using the ARCHITECH HBsAg, anti-HBs, HBeAg, and anti-HBe reagent kit (Abbott GmbH & Co., KG., Wiesbaden, Germany), respectively.

### Assessment of renal function

BUN and sCr was measured using an automatic analyzer (Hitachi 7170A, Hitachi Ltd., Tokyo, Japan). The eGFR was estimated using the following formulas based on age and sCr as previously described (Zhang et al., 2017). The Chronic Kidney Disease Epidemiology Collaboration (CKD-EPI) calculation for eGFR (mL/min/1.73 m^2^) = 141 × min (sCr/κ, 1)^α^ × max (sCr/κ, 1)^–1.209^ × 0.993*^age^* × 1.018 (if female). κ is 0.7 for female and 0.9 for male. α is −0.329 for female and −0.411 for male (Levey et al., 2009). The Modification of Diet in Renal Disease (MDRD) calculation for eGFR (mL/min/1.73 m^2^) = 186 × sCr^–1.154^ × age^–0.203^ × 0.742 (if female) (Levey et al., 1999).

### Statistical analysis

SPSS 23.0 was used for general statistical analysis. Shapiro-Wilk test was used for normal distribution assay. The variables following normal distribution were presented as mean ± standard deviation (SD), and the statistical significance was determined by one-way analysis of variance (ANOVA) followed by Tukey test. The variables following skewed distribution were presented as median (range). To evaluate the association between several variables and eGFR changes over time, a linear mixed effects model for repeated measures was used by SAS 9.4 with MIXED procedure. The model considered the baseline age (in years), gender, body weight, HBV DNA, HBsAg, BUN, sCr, and treatment strategy as fixed effects and incorporated random effects for individual subjects. All *P*-values are two-sided, and type I error was set as 5%.

## Results

### Characteristics of enrolled patients

A total of 114 CHB patients with PEG-IFNα2b add-on therapy were enrolled in this study. Baseline characteristics for patients were shown in Table 1. Fourteen (12.28%) CHB patients had detectable serum HBV DNA with low level viremia [89 (52∼712) IU/mL]. Baseline HBsAg level was 182.9 (0.13∼1295.37) IU/mL. Based on CKD-EPI formula, three patients showed an eGFR less than 90 mL/min/1.73 m^2^. Based on MDRD formula, seven patients revealed an eGFR less than 90 mL/min/1.73 m^2^. No patients showed a baseline eGFR less than 60 mL/min/1.73 m^2^ based on both formulas. All patients received at least 24-week PEG-IFNα2b therapy. Twenty two patients withdrew from PEG-IFNα2b therapy due to poor response (less than 10% of HBsAg down-regulation compared with baseline level) at 24 weeks of treatment, and 36 patients withdrew at 36 weeks of therapy. Fifty six (49.12%) patients completed 48-week PEG-IFNα2b add-on therapy.

**TABLE 1:** Baseline characteristics of enrolled patients.

Characteristic	Value
Patients enrolled, *n*	114
Male gender, *n* (%)	78 (68.42%)
Body weight (kg)	69.23 ± 13.18
Age, years, mean ± SD	41.49 ± 9.91
<40 years, *n* (%)	48 (42.11%)
≥40 years, *n* (%)	66 (57.89%)
HBV DNA undetectable (<50 IU/mL), *n* (%)	100 (87.72%)
HBV DNA detectable (>50 IU/mL), *n* (%)	14 (12.28%)
HBsAg, IU/mL, median (range)	182.9 (0.13∼1295.37)
HBsAg < 250 IU/mL, *n* (%)	63 (55.26%)
HBsAg > 250 IU/mL, *n* (%)	51 (44.74%)
HBeAg positive, *n* (%)	7 (6.14%)
HBeAg negative, *n* (%)	107 (93.86%)
BUN, mmol/L, mean ± SD	4.57 ± 1.13
sCr, μmol/L, mean ± SD	65.60 ± 12.07
eGFR (CKD-EPI), mL/min/1.73 m^2^, mean ± SD	111.0 ± 12.46
eGFR (MDRD), mL/min/1.73 m^2^, mean ± SD	116.9 ± 22.22

SD, standard deviation; HBsAg, Hepatitis B surface antigen; HBeAg, Hepatitis B e antigen; BUN, blood urea nitrogen; sCr, serum creatinine; eGFR, estimated glomerular filtration rate; CKD-EPI, Chronic Kidney Disease Epidemiology Collaboration; MDRD, Modification of Diet in Renal Disease.

### Changes in renal function in response to pegylated interferon-α2b add-on therapy

**FIGURE 1:**
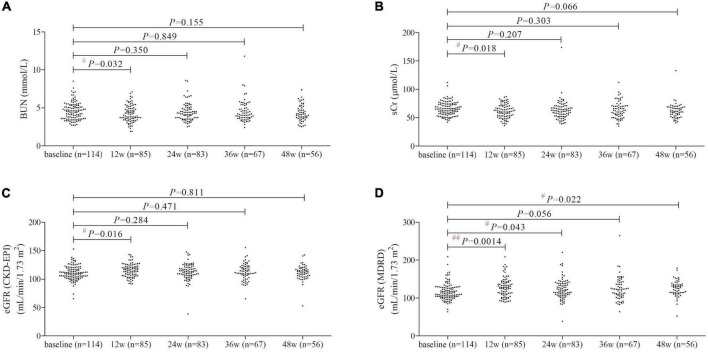
Evolution of renal function by PEG-IFNα2b add-on therapy over 48 weeks. **(A)** Change of BUN. **(B)** Changes of sCr. **(C)** Changes of eGFR as calculated by CKD-EPI formula. **(D)** Changes of eGFR as calculated by MDRD formula. Individual level for each value was shown. Statistical analysis was performed using one-way ANOVA followed by Tukey test.

BUN and sCr level was reduced at 12 weeks of therapy (*P*< 0.05), but there were no significant differences of either BUN or sCr level at 24, 36, or 48 weeks of therapy compared with baseline (*P*> 0.05, Figures 1A,B). Based on CKD-EPI formula, eGFR was increased at 12 weeks of therapy compared with baseline (+4.2 mL/min/1.73 m^2^, *P* = 0.016, Figure 1C), but did not remarkably change at 24, 36, or 48 weeks of therapy (*P*> 0.05, Figure 1C). One patient had lowest eGFR CKD-EPI level (38.54 mL/min/1.73 m^2^) at 24 weeks of therapy whose baseline level was 65.64 mL/min/1.73 m^2^, and eGFR CKD-EPI level was 52.95 mL/min/1.73 m^2^ at 48 weeks of therapy. Based on MDRD formula, there was maintenance of eGFR improvement during 48 weeks of PEG-IFNα2b add-on treatment (Figure 1D). eGFR MDRD reached peak level at 12 weeks of therapy (127.9 ± 25.49 mL/min/1.73 m^2^, *P* = 0.0014, Figure 1D), and maintained at 125.1 ± 21.00 mL/min/1.73 m^2^ at 48 weeks of treatment (*P* = 0.022, Figure 1D). Based on the MDRD formula, two patients had eGFR less than 90 mL/min/1.73 m^2^ at 48 weeks of therapy.

### Predictors of significant renal function changes in response to pegylated interferon-α2b add-on therapy

**FIGURE 2:**
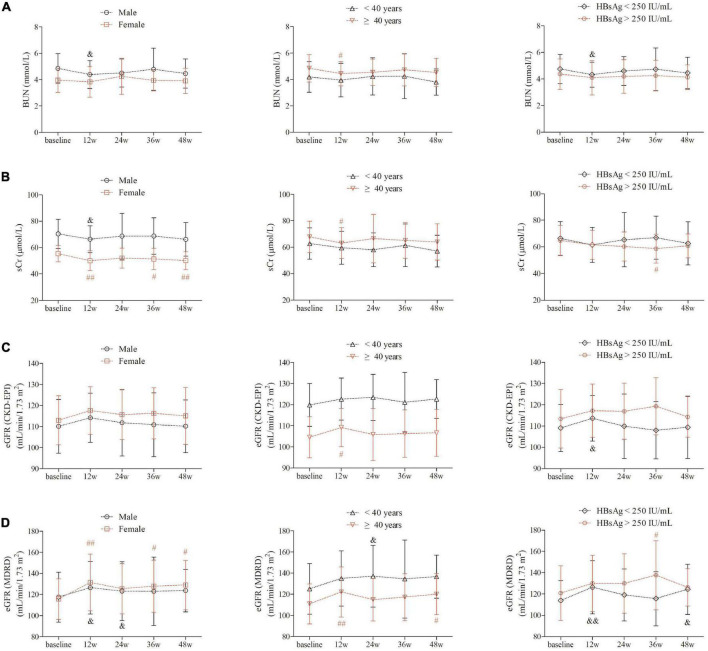
Evolution of renal function by PEG-IFNα2b add-on therapy over 48 weeks under different factors. **(A)** Change of BUN between different gender, between different age (<40 years and ≥40 years), and between different baseline HBsAg level (<250 and >250 IU/mL). **(B)** Changes of sCr between different gender, between different age (<40 and ≥ 40 years), and between different baseline HBsAg level (<250 and >250 IU/mL). **(C)** Changes of eGFR as calculated by CKD-EPI formula between different gender, between different age (<40 and ≥40 years), and between different baseline HBsAg level (<250 and >250 IU/mL). **(D)** Changes of eGFR as calculated by MDRD formula between different gender, between different age (<40 and ≥40 years), and between different baseline HBsAg level (<250 and >250 IU/mL). Statistical analysis was performed using one-way ANOVA followed by Tukey test. ^&^ and ^#^indicates *P* < 0.05 compared with baseline. ^&&^ and ^##^indicates *P* < 0.01 compared with baseline.

BUN level revealed statistical down-regulation at 12 weeks of therapy in male gender, elder patients, and patients with baseline HBsAg less than 250 IU/mL (*P*< 0.05, Figure 2A). sCr level was significantly decreased in female gender at 12 weeks of therapy, and maintained in lower lever at 36 and 48 weeks of therapy in female gender (Figure 2B). However, sCr level was only statistically reduced at 12 weeks of therapy in male gender (*P* = 0.028, Figure 2B) and in patients with age ≥ 40 years (*P* = 0.039, Figure 2B). Based on CKD-EPI formula, eGFR level did not remarkably change over time in either genders (*P*> 0.05, Figure 2C). eGFR CKD-EPI level was increased at 12 weeks of therapy in patients with age ≥ 40 years (*P* = 0.012, Figure 2C) and in patients with baseline HBsAg less than 250 IU/mL (*P* = 0.034, Figure 2C). Importantly, based on MDRD formula, eGFR was robustly elevated at 12 weeks of therapy, and was maintained in higher level in female gender, patients with age ≥40 years and in patients with baseline HBsAg less than 250 IU/mL (*P*< 0.05, Figure 2D).

Furthermore, we entered all variables as fixed effects and incorporated random effects in the linear mixed model accounting for repeated measures. Previous studies have been demonstrated that ETV therapy was not associated with the either improvement or deterioration of renal function in CHB patients (Park et al., 2017; Zhang et al., 2017; Suzuki et al., 2019), ETV monotherapy in 44 CHB patients who were continuously monitored renal function over 48 weeks was set as reference in this model. Results with CKD-EPI and MDRD equation showed comparable predictor value for eGFR changes at 24 weeks of PEG-IFNα2b add-on therapy. Female gender, baseline detectable HBV DNA, sCr, and PEG-IFNα2b add-on were significant predictors for increase eGFR (Table 2). At 48 weeks of therapy, female gender was also a positive predictor for eGFR MDRD. Female gender also revealed an estimated value of 4.135 for eGFR CKD-EPI, but it failed to achieve statistical difference (*P* = 0.075, Table 3). Baseline sCr and PEG-IFNα2b add-on were observed to be positively affected eGFR values at 48 weeks of therapy (all *P*< 0.001, Table 3). However, the changes of eGFR was not remarkably associated with body weight, age, baseline HBsAg level, or baseline BUN (*P*> 0.05, Tables 2, 3).

**TABLE 2:** Predictors for eGFR changes at 24 weeks of therapy.

	eGFR (CKD-EPI)			eGFR (MDRD)		
	Estimate	Standard error	*P-*value^a^	Estimate	Standard error	*P*-value^a^
Female gender	3.915	1.690	0.021	11.553	4.740	0.015
Body weight	–0.076	0.056	0.169	–0.074	0.143	0.603
Age	–0.043	0.066	0.516	–0.146	0.178	0.412
HBV DNA > 50 IU/mL (baseline)	3.065	1.132	0.007	8.022	3.208	0.012
HBsAg level > 250 IU/mL (baseline)	–0.002	0.003	0.397	–0.008	0.007	0.253
BUN (baseline)	–0.282	0.549	0.608	–0.574	1.465	0.695
sCr (baseline)	0.218	0.075	0.004	0.418	0.206	0.043
PEG-IFNα2b add-on	3.655	1.532	0.017	9.413	4.032	0.020

^a^Results from the linear mixed effects model for repeated measures.

**TABLE 3:** Predictors for eGFR changes at 48 weeks of therapy.

	eGFR (CKD-EPI)			eGFR (MDRD)		
	Estimate	Standard error	*P*-value^a^	Estimate	Standard error	*P*-value^a^
Female gender	4.135	2.326	0.075	17.090	5.055	0.0007
Body weight	–0.101	0.057	0.077	–0.153	0.115	0.183
Age	0.046	0.072	0.519	0.149	0.187	0.425
HBV DNA > 50 IU/mL (baseline)	2.268	1.311	0.084	2.505	3.294	0.447
HBsAg level > 250 IU/mL (baseline)	–0.000	0.0001	0.688	0.000	0.0001	0.998
BUN (baseline)	0.561	0.711	0.430	1.369	1.647	0.406
sCr (baseline)	0.432	0.091	<0.0001	1.078	0.207	<0.0001
PEG-IFNα2b add-on	6.780	1.559	<0.0001	14.949	3.896	0.0001

^a^Results from the linear mixed effects model for repeated measures.

## Discussion

Chronic HBV infection robustly elevated the risk of end-stage renal diseases (Chen et al., 2015). Inhibition of HBV replication by antiviral agents could decrease HBsAg level, leading to the reduction or even clearance of HBV antigenemia and further improvement of renal impairment in patients with hepatitis B related-glomerulonephritis (Liu et al., 2020). However, antiviral therapy might also result in deterioration of kidney injury due to the nephrotoxicity of drugs (Liu and Kao, 2019). Herein, we designed to assess the renal function of CHB patients who received PEG-IFNα2b (Y shape, 40 kD) add-on therapy to ETV. The important finding was that eGFR improved remarkably in response to PEG-IFNα2b combined with ETV treatment over 48 weeks. While ETV therapy showed dispensable effect to renal function (Zhang et al., 2017), PEG-IFNα2b might mainly contribute to the renal protective effect during the combination therapy. Interestingly, female gender was proven to be an important positive predictive factor for eGFR changes during PEG-IFNα2b plus ETV therapy under both one-way ANOVA analyses and linear mixed effects model for repeated measures. Collectively, PEG-IFNα2b add-on therapy might closely associate with renoprotective effect for CHB treatment.

Controversy remained as to the IFNα monotherapy or combination with NAs therapy to renal function. Recombinant IFNα2b improved immune response to hepatitis B vaccination in hemodialysis patients (Miquilena-Colina et al., 2009), indicating the safety profile of IFNα2b to patients with renal dysfunction. Renal allograft recipients who had chronic hepatitis B, C, and D were treated with IFNα three times weekly for 6 months. Renal allograft function remained stable in 31 patients (73.81%) during IFNα therapy, and the antiviral efficiency was only mild to moderate during long-term followed-up (Durlik et al., 1998). IFNα was not recommended for renal allograft recipients with hepatitis virus infection (Durlik et al., 1998). eGFR level was notably lower in patients receiving ADV therapy compared with PEG-IFNα2a treatment in chronic hepatitis B/D co-infection (Mederacke et al., 2012). Importantly, Combination treatment of PEG-IFNα2a plus ADV did not lead to further renal dysfunction (Mederacke et al., 2012), indicating the potential renal protective effect of PEG-IFNα2a, which might counteract the nephrotoxicity of ADV. The current finding of the renoprotective effect of PEG-IFNα2b (Y shape, 40 kD; 180 μg) add-on therapy was consistent with our previous reports on PEG-IFNα2a (U shape, 40 kD; 180 μg) monotherapy in both treatment-naïve and ETV-experienced CHB patients (Zhang et al., 2017), however, was not in line with the findings of PEG-IFNα2b (line shape, 12 kD; 1.5 μg/kg) monotherapy and combined with ADV treatment in CHB patients, which showed the improvement of renal function at 4 weeks of therapy but steadily declined over 48 weeks (Su et al., 2018). In our opinions, the differential effect of PEG-IFNα2b (Y shape, 40 kD; 180 μg) and PEG-IFNα2b (line shape, 12 kD; 1.5 μg/kg) might mainly due to the different molecule weight of PEG and branched monomethoxy PEG (Monfardini et al., 1995). The branched PEG (Y shape and U shape) demonstrated higher molecule weight than linear PEG (40 kD vs. 12 kD). Moreover, the proteins modified by branched PEG revealed not only elevated *in vitro* activity and proteolytic resistance, but also improved stability toward temperature and pH variations as well as enhanced half-life (Monfardini et al., 1995). Thus, PEG-IFNα2a (U shape, 40 kD) and PEG-IFNα2b (Y shape, 40 kD) could maintain higher concentration in peripheral blood than PEG-IFNα2b (line shape, 12 kD). Both high molecule weight and branched shape might contribute to the significant reduction in renal excretion ratio of drugs, resulting in the potential renoprotective priority of branched PEG modified IFNα. Furthermore, it is generally elucidated that deposition of immune complexes of HBV antigens and host antibodies mediate glomerular injuries. Peripheral CD4^+^CXCR5^+^ follicular T helper (Tfh) cell frequency was negatively correlated with the value of eGFR (Liu et al., 2014), suggesting that Tfh might contribute to IFN-induced improvement of eGFR. However, the specific mechanisms by which PEG-IFNα2b (Y shape, 40 kD) exert the renoprotective effects remains to be clarified.

We then investigated the potential indicators associated with eGFR changes over time. Our previous study revealed that age and baseline BUN were significant negative predictors for eGFR changes in CHB patients receiving either PEG-IFNα2a or NAs therapy (Zhang et al., 2017). Su et al. (2018) showed that age, baseline HBV DNA, and ADV-containing therapy are important predictable factors for eGFR decrease in patients with PEG-IFNα2b therapy, which was similar to the findings by Mederacke et al. (2012) in chronic hepatitis B/D co-infection. Herein, we analyzed the potential predictors for eGFR changes in response to PEG-IFNα2b (Y shape, 40 kD) add-on therapy to ETV *via* two different statistical methods. On the one hand, we compared the changes renal function over time between different genders, ages, and baseline HBsAg level using one-way ANOVA. We found that patients with female gender, age ≥ 40 years, and baseline HBsAg level < 250 IU/mL showed significant improvement of renal function with PEG-IFNα2b add-on therapy. However, we did not involve baseline HBV DNA level as one of the potential factors due to the limited cases with detectable HBV DNA (*n* = 14). On the other hand, we entered all baseline variables as fixed effects and incorporated random effects in a linear mixed model accounting for repeated measures. The results showed that female gender, baseline sCr, and PEG-IFNα2b add-on were significant positive predictors for eGFR elevation at 24 and 48 weeks of therapy. Although detectable HBV DNA was the positive indicator for eGFR changes at 24 weeks, it failed to achieve statistical significance at 48 weeks of therapy. Collectively, female gender might be the most important positive predictor for renoprotection during PEG-IFNα2b (Y shape, 40 kD) add-on therapy.

There were several limitations of the present study. Firstly, this was a retrospective analysis of renal function in a real-world setting. The followed-up data were not complete in several patients. We collected the data in at least three observation time points to reduce errors and bias. The large scale prospective study with longer observational time (both during and post treatment) are needed to confirm the current findings. Secondly, NAs might be harmful to both glomerular and tubular cells in the kidney (Kayaaslan and Guner, 2017; Wong et al., 2018). The mechanisms and target cells of PEG-IFNα2b (Y shape, 40 kD) for renoprotective effect were still unclear.

## Conclusion

In summary, our present results provided the evidence that PEG-IFNα2b (Y shape, 40 kD) add-on treatment might contribute to the increased eGFR for CHB patients in real-world experience. The mechanisms underlying the beneficial effects remained to be further clarified.

## Data availability statement

The raw data supporting the conclusions of this article will be made available by the authors, without undue reservation.

## Ethics statement

The studies involving human participants were reviewed and approved by the Ethics Committee of Tangdu Hospital and Ethics Committee of the Third People’s Hospital of Taiyuan. The patients/participants provided their written informed consent to participate in this study.

## Author contributions

YZ and YG contributed to study concept, design, and manuscript revision. M-JP, X-QG, JC, WK, and X-FY contributed to data acquisition. M-JP, W-LZ, YG, and YZ contributed to data analysis. M-JP, X-QG, W-LZ, and JC contributed to manuscript drafting. All authors approved the final version of the manuscript.
